# Developmental Outcomes of Infants Treated with Combination Therapy for Infantile Spasms

**DOI:** 10.15844/pedneurbriefs-33-2

**Published:** 2019-12-31

**Authors:** Jacqueline J. Wolak, Sunita N. Misra

**Affiliations:** 1 Division of Neurology and Epilepsy, Ann & Robert H. Lurie Children’s Hospital of Chicago, Chicago, IL; 2 Department of Pediatrics, Northwestern University Feinberg School of Medicine, Chicago, IL

**Keywords:** Infantile Spasms, Epilepsy, ACTH, Vigabatrin, Combination Therapy

## Abstract

Researchers at the International Collaborative Infantile Spasms Study (ICISS) conducted a study to assess the developmental and epilepsy outcomes in infants treated with combination (hormonal and vigabatrin) therapy for the diagnosis of infantile spasms.

Researchers at the International Collaborative Infantile Spasms Study (ICISS) conducted a study to assess the developmental and epilepsy outcomes in infants treated with combination (hormonal and vigabatrin) therapy for the diagnosis of infantile spasms. Hormonal therapy was either adrenocorticotropin (ACTH) or prednisolone. A total of 377 infants with infantile spasms and a hypsarhythmic (or similar) electroencephalogram (EEG) were randomly assigned to combination therapy (n = 186) and hormonal therapy (n = 191). Developmental and epilepsy outcomes were evaluated at 18 months blinded to treatment, with 181 infants in each treatment group. Analysis was completed by intention to treat.

The Vineland Adaptive Behavior Scale (VABS) was used to measure the difference in developmental outcomes at 18 months. The mean VABS composite scores showed no significant difference in developmental outcomes between both treatment groups (73.9, standard error [SE] 1.3 vs. 72.7 , SE 1.4; difference –1.2, 95% confidence interval [CI] –4.9 to 2.6; p = 0.55). There was also no significant difference in the presence of epilepsy reported by caregivers or as a result of antiepileptic treatment (including ketogenic diet) in the previous 28 days between both treatment groups (54 infants [30.0%]: combination therapy vs. 52 infants [29.2%]: hormonal therapy; difference 0.8% [95% CI –8.8 to 10.4]; p = 0.90); there was also no significant difference in the presence of infantile spasms between both treatment groups (27 infants [15.0%]: combination therapy vs. 28 infants [15.7%]: hormonal therapy; difference 0.7% [95% CI –6.9 to 8.3];p = 0.85).

This study also reported that spasm cessation between days 14 and 42 of the treatment was associated with higher mean VABS scores and reduced probability of seizures at 18 months. In addition, delay to treatment time was associated with lower VABS scores and poorer epilepsy outcomes (p = 0.023). [1]

COMMENTARY. This study concluded that there was no significant change in developmental or epilepsy outcomes at 18 months for infants treated with combination therapy versus hormonal therapy alone. However, the previous study by ICISS reported that combination therapy was more effective than hormonal therapy alone in treating spasms between days 14 and 42 of treatment and that early spasm cessation is a strong predictor of epilepsy outcomes [2]. Acknowledging these improved treatment outcomes, combination therapy should be preferred over hormonal therapy alone in most clinical cases. This study supports the fact that early treatment of infants with infantile spasms is associated with improved epilepsy and developmental outcomes [3].

Infants treated with hormonal monotherapy who did not respond well were quickly given vigabatrin, which is an effective combination therapy that may enhance developmental and epilepsy outcomes at18 months. Evaluation of etiological causes for infants with infantile spasms in each cohort may also be informative and help identify subgroups that are more sensitive to combination therapy.

In conclusion, this study supports both rapid and effective treatment of infantile spasms.

## Disclosures

The authors have declared that no competing interests exist.
